# Data of the properties of rebar steel brands in Lagos, Nigerian market used in reinforced concrete applications

**DOI:** 10.1016/j.dib.2018.01.083

**Published:** 2018-02-03

**Authors:** Opeyemi Joshua, Kolapo O. Olusola, Kehinde D. Oyeyemi, Ayodeji O. Ogunde, Lekan M. Amusan, David O. Nduka, Joyce Abuka-Joshua

**Affiliations:** a Department of Building Technology, Covenant University, Ota, Nigeria; b Department of Building, Obafemi Awolowo University, Ile-Ife, Nigeria; c Department of Physics, Covenant University, Ota, Nigeria

## Abstract

The data presented herein are compilations of the research summary of “Assessment of the Quality of Steel Reinforcement Bars Available in Nigerian Market” (Joshua et al., 2013) [1]. This data article provides information on the properties and cost of steel rebars used in reinforced concrete in Lagos, Nigeria. The data is based on the properties of 12 mm rebar brands which are the most used steel diameter in construction and they include actual diameters, yield strengths, ultimate strengths, ultimate/yield strength ratio, ductility and the cost of each brand. This data also contains the limiting standard properties of the highlighted properties in this data.

**Specifications Table**

**Table:** 

Subject area	Engineering, Material Science and Concrete
More specific subject area	Reinforced Concrete, Construction and Civil Engineering
Type of data	Figures and Bar Charts
How data was acquired	The data was obtained from standard laboratory procedures and analyzed with simple statistical tools and market survey
Data format	Raw and Analyzed
Experimental factors	Nine (9) steel brands (A-J) were analyzed and tests such as tensile yield and ultimate strengths and ductility were conducted. A survey on the cost of each brand was conducted also.
Experimental features	Physical and mechanical laboratory tests alongside market survey of their prices.
Data source location	Nigeria. West Africa
Data accessibility	The data are available within this article
Related research article	O. Joshua, K.O. Olusola, C. Ayegba and A. Yusuf, A. I. “Assessment of the Quality of Steel Reinforcement Bars Available in Nigerian Market”, Conference Proceedings of the Architectural Engineering Institute (AEI) of the American Society of Civil Engineers (ASCE). 2013.

**Value of the data**

• This data will assist in assessing the role of steel rebars in the quality of our reinforced concrete properties and form a basis on how to prefer solutions to managing the challenges the state of the steel might pose to construction and durability of building structures.
• The data can be used in collaboration with other related research outcome to develop a local factor of safety for the steel rebar as used in the design of reinforced concrete.
• This data can form a basis for the standard regulatory bodies or agencies to query erring steel rebar manufacturing brands with a view to improving their quality or appraising complying brands.

## 1 Data

**Fig. 1:**
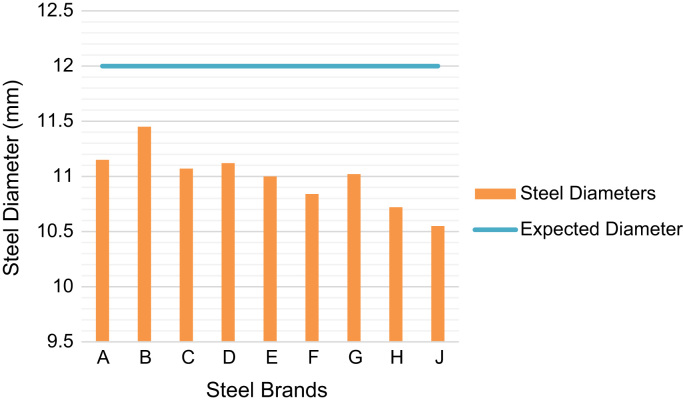
Actual Diameters of steel brands.

**Fig. 2:**
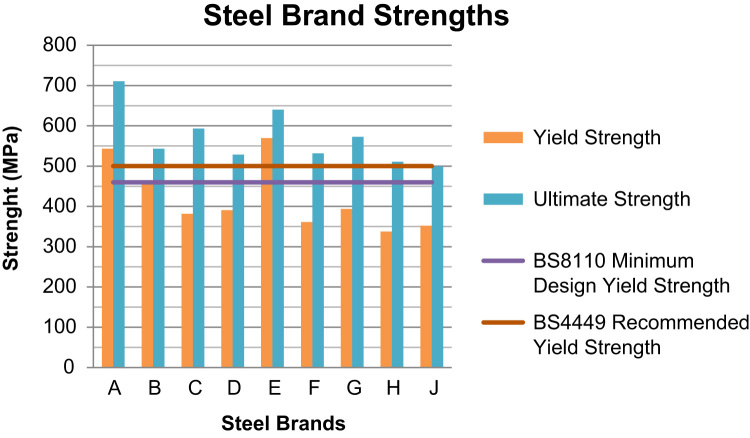
Steel Brand's yield and ultimate strengths.

**Fig. 3:**
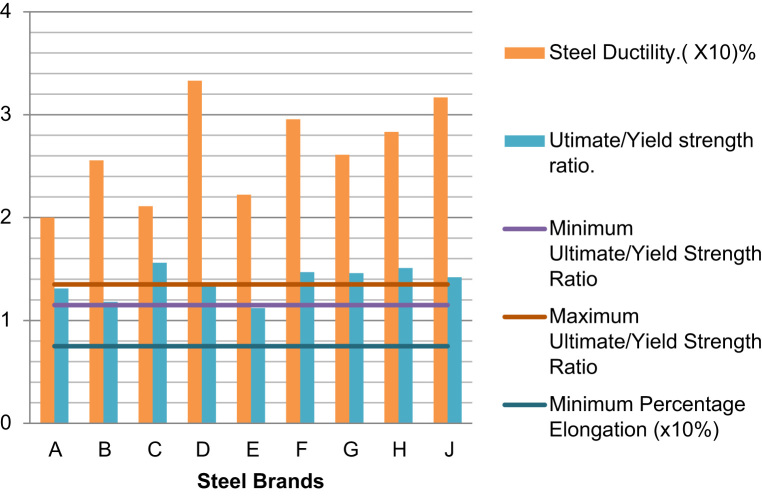
Ductility and Ultimate/Yield strength ratio of different steel brands.

**Fig. 4:**
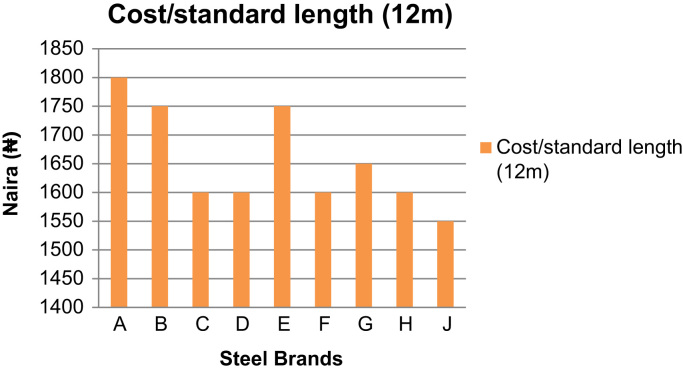
Cost of standard length (12 m) of the different steel brands.

Over 90% of storey building in Nigeria are of structures made from reinforced concrete [1], [2], where all tensile stresses in these concrete structures are resisted by the steel rebars embedded in the concrete. These steel rebars are designed on the assumptions that they possess right cross-sectional diameters and areas, they are of minimum tensile strengths of 460 MPa as specified in the design standards, and they are of specific elongation (ductility) to prevent sudden failures [3]. If the rebars available in the market used in construction possess properties less than these design assumptions, then the failures of such structures become inevitable before their expected lifespan. The data presented herein (Fig. 1, Fig. 2, Fig. 3, Fig. 4) were obtained from the physical and mechanical tests of the various steel rebar brands obtained from market survey of nine (9) brands of steel. The data also highlight the limiting standard properties obtained from standards regulating the steel rebars and design assumptions [3], [4].

## 2 Experimental design, materials, and methods

The method of sampling these rebars is by market survey to identify the steel brands used in this data, purchased three cuts of 12 mm diameter rebars for each identified steel brand from three different merchants. The cuts were labeled and sent to the laboratory for tests. 12 mm diameter bar size was chosen because it is the most used in structural design work in Nigeria [5]. Nine (9) steel brands were identified denoted as Brands A-J. The tests conducted include tensile yield and ultimate strengths, elongation (ductility), and prices per standard market length of the identified brands were also surveyed. The tests performed are in triplicates and the average used as represented in this data. Lesser rebar diameter than specified indicate less cross-sectional area of the rebars in reinforced concrete and hence lesser tensile or compressive resistant strength characteristics. This is vice versa for larger diameters than the specified are used.

Reinforced concrete is designed according to a recommended standard procedure [3], which specifies a minimum of 460 MPa yield strength. The strength of steel rebars used to reinforce concrete is based on the assumption that if the actual yield strength is less than this minimum requirement, then the reinforced concrete may lose the capacity to effectively resist the tensile service loads. This translates to a reduced strength of the structure. Ductility in steel is a desired property for occupants’ safety in reinforced concrete structures. In the event of reinforced concrete failures, especially under flexural loads, concrete reinforced with ductile steels give warnings like excessive beam deflection which is actually a precursor to the total failure of the structure. The occupants of such structures are therefore given sufficient time to evacuate before the total collapse [1]. Several works on the factors mitigating against construction projects in developing countries have been carried out [6], [7].
